# Photochemically Immobilized 4-Methylbenzoyl Cellulose as a Powerful Chiral Stationary Phase for Enantioselective Chromatography

**DOI:** 10.3390/molecules21121740

**Published:** 2016-12-17

**Authors:** Eric Francotte, Dan Huynh, Tong Zhang

**Affiliations:** Novartis Pharma AG, Global Discovery Chemistry, 4002-Basel, Switzerland; dan.huynh@novartis.com

**Keywords:** enantioselective chromatography, chiral stationary phase, immobilized methylbenzoyl cellulose, photochemistry

## Abstract

A process to immobilize *para*-methylbenzoyl cellulose (PMBC) on silica gel has been developed and applied to prepare chiral stationary phases (CSPs) for enantioselective chromatography. The immobilization was achieved by simple irradiation of the polysaccharide derivative with ultraviolet light after coating on a silica gel support. The influence of parameters such as irradiation time and solvent on immobilization effectiveness were investigated. The performance of the prepared immobilized phases were evaluated by injection of a series of racemic compounds onto the packed columns and determination of their chiral recognition ability. By contrast to the classical coated phase, the immobilized CSP can be used under various chromatographic conditions without limitation of organic solvent types as the mobile phase. This extended applicability permits to improve selectivity and to resolve chiral compounds which are not or only poorly soluble in the mobile phases which are compatible with the non-immobilized PMBC stationary phase.

## 1. Introduction

Polysaccharide-based chiral stationary phases (CSPs), especially those derived from cellulose and amylose derivatives, have proved to be versatile and effective means for enantioseparations by high performance liquid chromatography (HPLC) and supercritical fluid chromatography (SFC) [1,2]. They cover a large scope of applications on either analytic or preparative scale. Originally these CSPs were prepared as a coating by physical adsorption of the polysaccharide derivatives on a silica gel support [3,4,5,6] or as a pure polymer in the form of beads [7,8]. Over the last decade, a second generation of polysaccharide-based CSPs has appeared, consisting of the immobilized versions of the previously coated phases. This second generation of phase shows the unique advantage of being more stable and tolerating almost all kind of mobile phases by contrast to the ‘classical’ non-immobilized polysaccharide-based CSPs which readily dissolve in organic solvents, such as methylene chloride, chloroform, tetrahydrofuran, etc. This feature of the non-immobilized polysaccharide CSPs was a considerable limitation for analytes which exhibit low solubility, in particular for preparative applications.

Several techniques have been developed to immobilize polysaccharide derivatives on silica gel and these different techniques have been reviewed in recent papers [9,10,11]. Okamoto and his group was particularly active in this field and carried out already in 1987 the immobilization of cellulose tris(3,5-dimethylphenylcarbamate) and tris(3,5-dichlorophenylcarbamate) on 3-aminopropyl silica gel using a di-isocyanate moiety as the linking component [12]. Other groups developed processes to immobilize polysaccharide derivatives on silica gel. For example, Oliveros at al. performed the immobilization of polysaccharide derivatives through a radically initiated polymerization of a 10-undecenoyl group which was incorporated on the polysaccharide backbone [13]. We also developed a technique of immobilization consisting in a photochemical or thermal treatment of the polysaccharide derivatives after coating on silica gel [14,15,16,17]. In this report we show that this approach is very simple and does not need the relatively tedious operation of selective protection and deprotection chemistry used in most of the other immobilization approaches, as no attaching or crosslinking group is introduced onto the polysaccharide chain. The simplicity of the method as well as the influence of various parameters on the immobilization effectiveness is exemplified with the application to *para*-methylbenzoyl cellulose (PMBC).

## 2. Results and Discussion

### 2.1. Preparation of the Immobilized PMBC-Im Stationary Phases (CSP)

**Figure 1:**
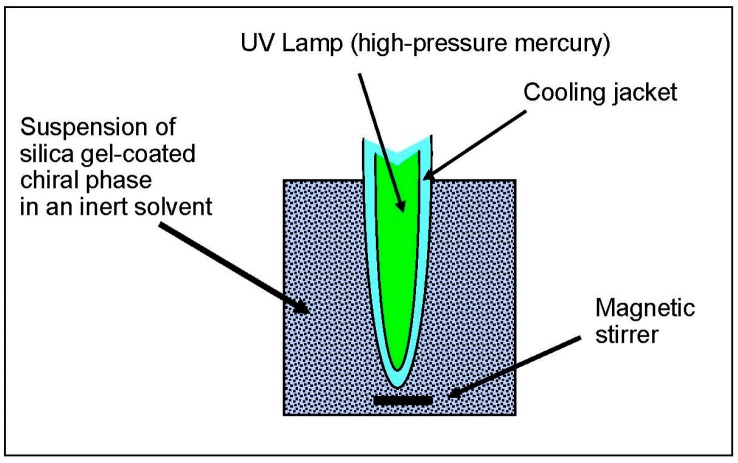
Schematic photochemical setup for immobilization of PMBC on silica gel.

Immobilization of PMBC after coating on silica gel was achieved by irradiation of a suspension of the coated material in an inert solvent for a few hours, using an immerged mercury lamp surrounded by a Pyrex cooling jacket (Figure 1). Acetonitrile, or mixtures of methanol-water were used as the photochemical suspension solvent. After photochemical treatment, each sample was extracted with a mixture of chloroform, dichloromethane and tetrahydrofuran in order to remove the non-bonded parts of PMBC (see experimental part in Section 3.3 for more details).

Table 1 summarizes the preparation conditions and the characterization of the prepared immobilized PMBC-Im CSPs. The table indicates the CSP name, the amount of coated PMBC, the solvent mixture for irradiation, the irradiation time, the amount of immobilized material after irradiation and extraction treatment, and the immobilization rate.

In addition, a column packed with the coated material with no further treatment (no immobilization) has also been prepared as a reference (PMBC-S). This column has obviously not been used with mobile phases likely to dissolve the coated polysaccharide derivative PMBC.

Immobilization rate refers to the final amount of the immobilized polymer compared to the initial amount of coated PMBC on silica gel. From the results in Table 1 it can be noticed that while an irradiation time of 15 h in 25% methanol in water affords about 70% immobilization (PMBC-Im A), increasing the irradiation above 20 h does not further increase notably the immobilization degree which remains almost unchanged around 87% in the same suspension medium (PMBC-Im B-E). Irradiation in 50% methanol (PMBC-Im F) or pure acetonitrile (PMBC-Im G) give slightly lower immobilization rates.

Interestingly, the immobilization occurs without any addition of radical initiator or photosensitizer. Physical and chemical methods have been applied to get some insight into the immobilization mechanism. However, the insolubility of the material considerably limits the choice of physical means to investigate the structure of the obtained stationary phase at the molecular level. Solid state NMR, IR, and Raman spectroscopy were applied but no significant differences were found between the material obtained before and after immobilization. UV-irradiation of *para*-methylbenzoyl cellobiose was used as a model for PMBC and was carried out in solution but no trace of oligomers could be detected, suggesting that the immobilization process implicates a polymer effect. A very low percentage of crosslinking is actually sufficient to lead to a tridimensional insoluble network. It is not yet clear whether the polysaccharide derivative is immobilized by cross-linking between the polymer chains or by bonding to the silica gel carrier. It is known that cellulose and cellulose derivatives can degrade under UV treatment, producing radicals which might further react intra- or intermolecularly, but the exact degradation pathway is usually not easy to evidence [18,19,20]. In these reports, it was also shown that the formation of radicals does not necessarily need the presence of a photoinitiator.

**Figure 2:**
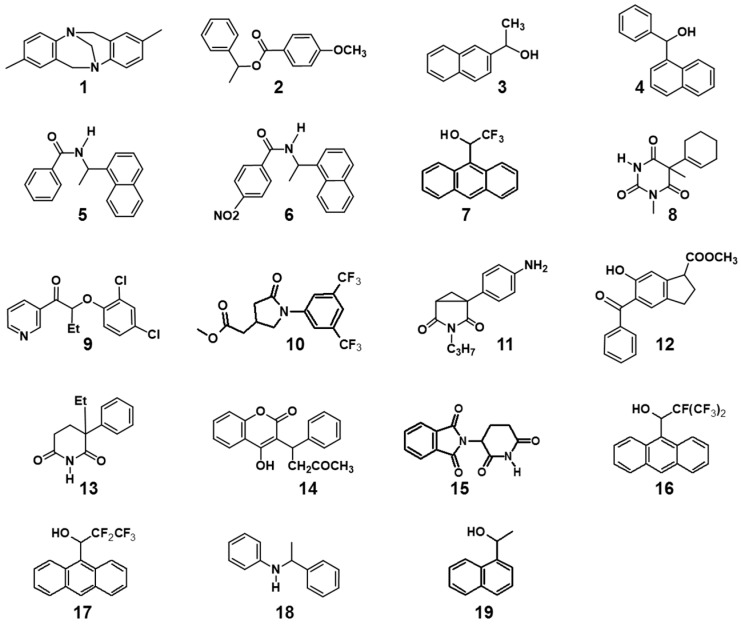
Structures of the tested racemic compounds.

The chiral recognition ability as well as the retention properties of the CSPs listed in Table 1 have been assessed using a series of racemic compounds. Figure 2 shows the structures of the selected racemic substances.

### 2.2. Influence of Irradiation Time on the Chromatographic Properties of the PMBC-Im CSPs

Selectivity and retention factors obtained for selected racemic solutes on the PMBC-Im CSPs prepared by irradiation in a mixture of methanol–water (25/75 in volume) after 18 h (PMBC-Im B), 24 h (PMBC-Im D), and 30 h (PMBC-Im E) are shown in Table 2. Results obtained on the non-immobilized phase PMBC-S are also shown as a reference. The classical mobile phase hexane/2-propanol (9/1, *v*/*v*) was used for all chromatographies.

Although there are some variations between the three immobilized CSPs in terms of selectivity and retention, most differences are relatively small. Selectivity values of the phases obtained after 18 h and 30 h irradiation respectively differ only in the range of 1%–4%. The difference for the retention factor values is more significant when comparing the same two CSPs, 4% to 13% depending on the compound, and might reflect the slightly higher amount of immobilized PMBC on the PMBC-Im E phase.

Compared to the non-immobilized phase PMBC-S, the negative impact of the immobilization on selectivity values is somewhat more marked especially for compounds **1**, **2**, and **9** but it remains minor for the other compounds. The small drop in selectivity indicates that the immobilization process partially disturbs the original structure of the cellulose derivative or restricts the access to the chiral interaction sites. However, for most substances the disturbance remains minor. This is probably due to the fact that, unlike other immobilization approaches, the designed process does not involve the introduction of specific functional immobilization groups onto the cellulose derivative, which alter the regularity of the molecular and supramolecular structure of the polysaccharide chains. The extent of selectivity decrease is not identical for each compound. It varies from 3% to 40% showing that each individual substance responses to the structural change of the polysaccharide differently. This observation is in accordance with previous investigations on cellulose triacetate which support the concept of the presence of multiple interaction sites in polysaccharide-based CSPs [21]. For the substances which exhibit a decrease of selectivity after immobilization using the conventional mobile phase hexane-isopropanol, the effect can in most instances be largely compensate by the utilization of mobile phases which are excluded with the non-immobilized stationary phase due to its solubility in these mobile phases (see Section 2.4). Interestingly, for the racemic substance **4** the selectivity is better on the immobilized phase.

### 2.3. Influence of Irradiation Solvent on the Chromatographic Properties of the PMBC-Im CSPs

It is known that photochemical reactions are dependent on solvent conditions and this effect has recently been reviewed by Klán et al. [22]. In order to investigate this parameter, we examined the effect of the suspension solvent on both immobilization rate and chiral recognition ability of the resulting immobilized CSPs PMBC-Im C, F, and G. For PMBC-Im C and F, 25% and 50% methanol in water was used respectively. For PMBC-Im G, pure acetonitrile was used as the solvent for irradiation of the suspended coated silica bead. 

As indicated in Section 2.1, the immobilization rate is somewhat affected by the type of used solvent. Increasing too much the methanol content seems to be less effective (PMBC-Im F) in terms of immobilization. This was confirmed by a test using pure methanol which afforded only 49% immobilization after 20 h. It has been reported that the stabilization of radicals can be considerably influenced by the polarity of the solvent, affecting the speed and the course of photochemical processes for small molecules [23,24,25]. However, this influence can be hardly predicted. Due to the insolubility of the immobilized composite prepared in this study and the unknown structure after immobilization of the cellulose derivative on silica gel, it is difficult to hypothesize about the exact influence of the solvent.

In terms of enantioselectivity, the three CSPs shows comparable results (Table 3) when hexane–isopropanol 9/1 (*v*/*v*) is used as the mobile phase, indicating that the intrinsic chiral recognition ability is identical for all phases and not much affected by the solvent conditions. 

There is no general trend for a CSP to be more performing, and again it depends on the racemic substance. For example, compounds **1**, **5**, and **13** are slightly better resolved on PMBC-Im F, while compound **2**, **7**, and **9** are slightly better resolved on PMBC-Im G. CSP PMBC-Im C shows generally higher retention properties (higher retention factors) but this probably due to the higher content of immobilized chiral material.

### 2.4. Modulating Enantioselectivity on Immobilized PMBC-Im CSPs with Mobile Phase Types and Composition

The unique feature of the immobilized polysaccharide-based phases is their ability to combine the extended chiral recognition ability of polysaccharide derivatives with the utilization of an unlimited range of mobile phases. This combination offers at least three major benefits, (i) the opportunity to achieve or improve enantioselectivity; (ii) the option to modulate retention time according to specific needs; (iii) the possibility to increase solubility of the racemic solute permitting to optimize productivity for preparative separations. 

These benefits are demonstrated by the numbers shown in Table 4 which summarizes the chromatographic data of PMBC-Im E for a variety of racemic compounds using mixtures of heptane and chloroform varying between heptane/chloroform 90/10 (*v*/*v*) and 60/40 (*v*/*v*) as the mobile phase. Chloroform was chosen because it is considered as a highly solvating agent for many poorly soluble compounds and possesses distinct properties in terms of selectivity. Values obtained on the same material before and after immobilization with hexane/2-propanol 90/10 as the mobile phase are also shown as a reference in Table 4. This mobile phase mixture is the usual mobile phase for the non-immobilized polysaccharide-based CSPs which do not tolerate chlorinated solvents.

**Figure 3:**
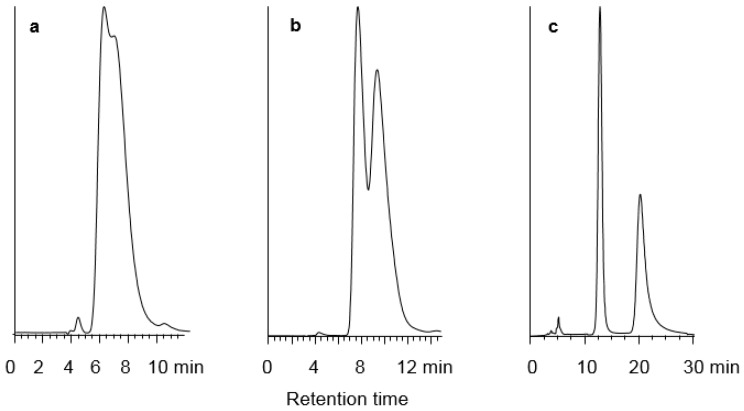
Chromatographic resolution of compound **16**. (**a**) on PMBC-S, mobile phase hexane/2-propanol 90/10 (*v*/*v*); (**b**) on PMBC-Im E, mobile phase hexane/2-propanol 90/10 (*v*/*v*); (**c**) on PMBC-Im E, mobile phase heptane/chloroform 75/25 (*v*/*v*).

Table 4 shows the selectivity values, retention factors (k_2_) and resolution obtained for 19 racemic compounds. Comparing the selectivity values of the CSPs PMBC-S and PMBC-Im E using hexane/2propanol 90/10 (*v*/*v*) as the mobile phase clearly shows that the selectivities systematically decrease after immobilization. The drop ranges between 2% and 15%, with one exception for compound **1** which shows a more significant decrease (25%) under the applied mobile phase conditions. However, for most of the racemic compounds, the selectivity could be recovered by using heptane-chloroform mixture as the mobile phase. For ten substances out of 15, the enantioselectivity was better on the immobilized CSP PMBC-Im E and for **4** compounds (**6**, **11**, **14**, **15**) the separation which could not be achieved on the non-immobilized phase due to the poor solubility in the mobile phase or excessive retention time (no elution) were easily resolved with heptane–chloroform mixtures as the mobile phases. An example of improved separation and selectivity is shown in Figure 3 for compound **16** when changing the mobile phase from hexane/2-propanol to heptane/chloroform.

**Figure 4:**
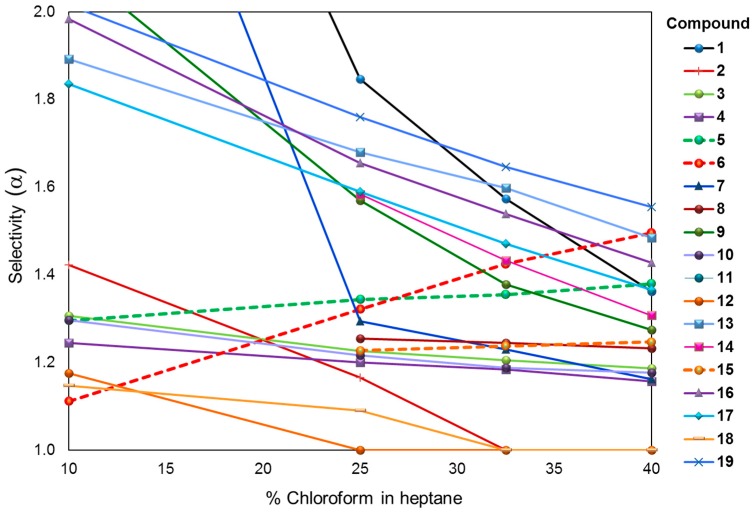
Chromatographic resolution of compound **1**–**19** on PMBC-Im E with heptane/chloroform as the mobile phase. Enantioselectivity vs. chloroform content in heptane.

**Figure 5:**
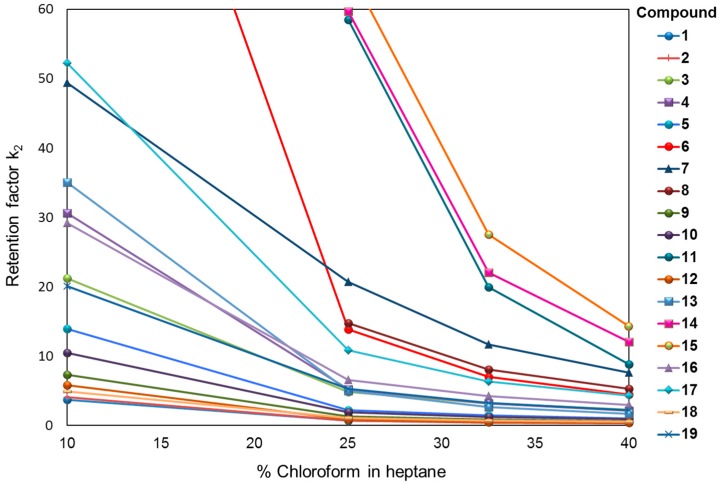
Chromatographic resolution of compounds **1**–**19** on PMBC-Im E with heptane/chloroform as the mobile phase. Retention factor k_2_ vs. chloroform content in heptane.

For compounds **8**, **11**, **14**, and **15** a higher content of chloroform is necessary to achieve the enantiomeric separation as retention is too long even with 10% chloroform in the mobile phase. Almost all compounds show a selectivity and retention decrease when the proportion of chloroform in heptane is increasing (Figure 4 and Figure 5).

**Figure 6:**
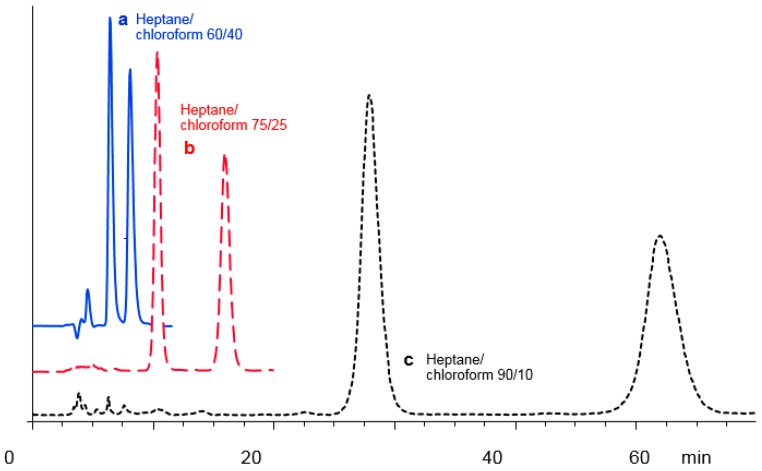
Chromatographic resolution of compound **19** on PMBC-Im E using heptane /chloroform mixtures as a mobile phase: (**a**) 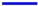 60/40 (*v*/*v*); (**b**) ----- 75/25 (*v*/*v*); and (**c**) **……** 90/10 (*v*/*v*).

For compounds **12** and **18** the separation is even lost above 30% of chloroform, indicating that for each enantiomeric pair the mobile phase composition has to be adjusted properly in order to keep selectivity while reaching a reasonable analysis time. They are three exceptions (compounds **5**, **6**, and **15**) for which selectivity is increasing with augmenting the proportion of chloroform (Figure 4). However, like for the other substances, the retention values are decreasing when the proportion of chloroform is increased (Figure 5). This effect might be extremely valuable for reducing analysis time as shown for compound **19** in Figure 6. 

Compound **6** exemplifies another benefit of the immobilized phase, namely the possibility to separate the enantiomers of substance which are not soluble in the conventional mobile phase (alkane–alcohol mixtures) for analysis or preparative scale resolutions. The compound is completely insoluble in hexane/2-propanol (9/1 *v*/*v*), but well soluble in mixture of heptane and chloroform. This solubility problem is often encountered in the practice.

**Figure 7:**
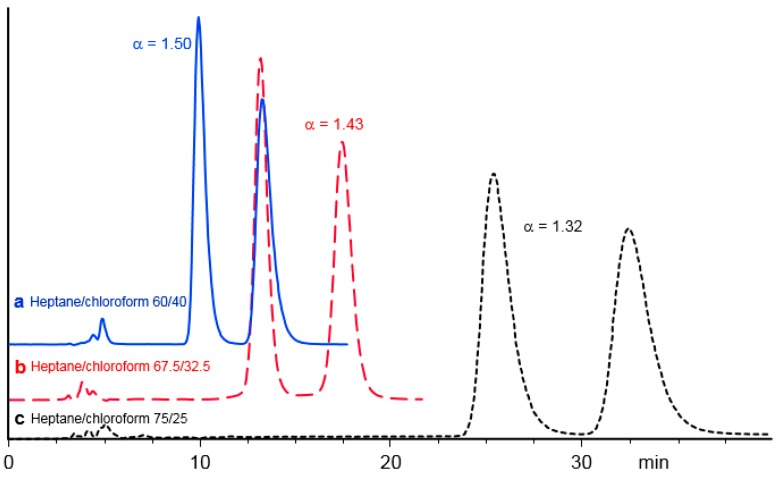
Chromatographic resolution of compound **6** on PMBC-Im E using heptane /chloroform mixtures as a mobile phase: (**a**) 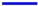 60/40 (*v*/*v*); (**b**) ----- 67.5/32.5 (*v*/*v*); and (**c**) **……** 75/25 (*v*/*v*).

For compounds **4**, **6**, **7**, **13** and **17** which exhibit very high retention factors and compounds **11**, **14**, **15** which are not eluted with heptane-chloroform 90/10 (*v*/*v*), increasing the chloroform content permits to considerably reduce the retention times, up to almost 30 times for substance **6**. This is of course of high value for analytical separations which are preferably performed at short retention times while maintaining a high enantioselectivity. This effect is illustrated for compound **6** on Figure 7.

For a few compounds (**2**, **12**, **18**) higher contents of chloroform cause a rapid elution of the two enantiomers which practically coelute at the dead time, leading to a loss of the separation.

## 3. Materials and Methods

### 3.1. Preparation of para-Methylbenzoyl Cellulose (PMBC)

*para*-Methylbenzoyl cellulose (PMBC) was prepared by reaction of microcrystalline cellulose (Avicel, Merck, Darmstadt, Germany) with an excess of 4-methylbenzoyl chloride in a mixture of pyridine and triethylamine in the presence of a catalytic quantity of 4-(dimethylamino)pyridine, as previously described [8]. 

### 3.2. Preparation Silica Gel Coated PMBC (PMBC-S)

Typically, a solution of 3.9 g PMBC dissolved in 100 mL dichloromethane is added to silica gel (10 g) (Nucleosil Si 4000-10, from Macherey-Nagel, Düren, Germany), which was preliminarily modified with 3-aminopropyltriethoxysilane. The mixture is stirred moderately for 30 min before evaporation of the solvent at 35 °C under reduced pressure (400 mbar) by means of a rotavapor. 

### 3.3. Preparation of Immobilized PMBC CSP (PMBC-Im)

After coating, 5.1 g of the obtained solid were suspended in 400 mL of a mixture of methanol and water or in pure acetonitrile. A high-pressure mercury-vapor lamp (125 W, HPK, Philips, Brussels, Belgium) with a Pyrex lamp housing was dipped into this suspension and turned on. The irradiation time was varied between 18 and 30 h. After photochemical treatment, the solution is filtered off and the obtained powder is washed with pure ethanol. The material is suspended for 24 h at room temperature in a mixture of 10 mL ethanol, 50 mL chloroform and 100 mL tetrahydrofuran in order to dissolve the non-bonded parts of the polymer. The solid phase is filtrated off and again washed with 100 mL chloroform and 100 mL THF. The dried material is finally suspended in a mixture of dichloromethane and chloroform (each 20 mL). An 8-fold volume of hexane was added dropwise to the suspension (1.6 mL/min) under moderate magnetic stirring. After completion of the addition of hexane, the suspension is filtrated off and the isolated solid is dried for 2 h at 120 °C. The final coating ratio, which is defined as the ratio of weight of the immobilized polymer on the total phase weight, was estimated by the recovered amount of methylene chloride-soluble PMBC and by elemental analysis. 

### 3.4. Column Packing of Immobilized PMBC CSPs

The material was packed into a stainless-steel column (250 × 4.6 mm I.D., Macherey-Nagel) by the slurry method using a mixture of hexane/ethanol (70/30 *v*/*v*) as a carrying liquid.

### 3.5. Equipment and Chromatographic Conditions

The HPLC system consisted of a LC-6A or LC-10 unit (Shimadzu, Kyoto, Japan) equipped with UV-visible detector and a 20-microliter sample loop. All chromatographic runs were performed at room temperature at a flow rate of 0.7 mL/min. The eluates were monitored at 254 nm. The dead time was determined by injecting 1,3,5-tri-tertbutylbenzene onto the column. 

### 3.6. Racemic Samples

Compounds **2**, **5**, **6**, **9**–**13**, **15**, and **19** were from Novartis. The synthesis of compounds **16** and **17** is described elsewhere [26]. Compounds **1**, **3**, **4**, **7**, **8**, and **14** were purshased from Sigma-Aldrich (Buchs, Switzerland).

## 4. Conclusions

Irradiation of coated PMBC phases with UV light constitutes a simple and efficient technique to immobilize para-methylbenzoyl cellulose on silica gel. The immobilized phase combines the advantages of the high chiral recognition ability of the cellulose derivative and the option to apply a wide range of mobile phase types, especially those which are not tolerated by the corresponding non-immobilized phase because of its high solubility in most organic solvents. It was shown that the extended range of applicable mobile phases can be exploited not only to separate the enantiomers of substances which are insoluble in the classical mobile phases but in many instances also to increase selectivity and to considerably reduce excessive retention times for strongly retained compounds. With the immobilized phase almost any kind of mobile phase is applicable, giving a highly extendable means to improve and adjust the chromatographic parameters such as retentiveness, enantioselectivity, and solubility of the solutes according to the intended purpose.

## Figures and Tables

**Table 1:** Preparation conditions and characterization of the immobilized PMBC-Im CSPs.

CSP Name	Amount of Coated PMBC (Weight %)	Irradiation Medium (Volume Ratio)	Irradiation Time (h)	Amount of Immobilized PMBC (Weight %)	Immobilization Rate (%)
PMBC-S	28	na	na	na	na
PMBC-Im A	28	25% MeOH in water	15	23.8	71.2
PMBC-Im B	28	25% MeOH in water	18	23.8	85.1
PMBC-Im C	28	25% MeOH in water	23	24.3	86.7
PMBC-Im D	28	25% MeOH in water	24	24.3	86.9
PMBC-Im E	28	25% MeOH in water	30	24.3	86.6
PMBC-Im F	28	50% MeOH in water	20	22	78.6
PMBC-Im G	25	100% acetonitrile	22	21.7	83.3

na: not applicable.

**Table 2:** Chromatographic results of the enantioselective separations of selected racemic compounds on photochemically immobilized PMBC-Im CSPs: influence of irradiation time in the mixture of methanol–water 25/75 (*v*/*v*). Mobile phase: hexane/2-propanol (9/1, *v*/*v*).

Compound	PMBC-S				PMBC-Im B				PMBC-Im D				PMBC-Im E			
	α	k_1_	k_2_	Rs	α	k_1_	k_2_	Rs	α	k_1_	k_2_	Rs	α	k_1_	k_2_	Rs
**1**	9.15	1.85	16.89	3.71	7.06	1.74	12.31	4.55	6.33	1.94	12.28	4.67	6.89	1.98	13.62	4.37
**2**	1.47	5.31	7.82	1.21	1.31	4.16	5.45	1.27	1.28	5.03	6.45	1.15	1.30	4.86	6.30	1.04
**4**	1.00	14.06	14.06	0	1.09	9.28	10.11	nd	1.00	10.44	10.44	0	1.12	9.38	10.50	0.86
**5**	1.30	9.98	13.01	0.65	1.25	10.12	12.63	0.89	1.24	11.4	14.13	0.75	1.22	11.29	13.80	0.66
**7**	1.22	7.63	9.35	nd	1.20	6.85	8.23	0.87	1.19	7.66	9.08	0.62	1.16	7.72	8.95	0.54
**9**	2.53	3.99	10.11	2.25	2.13	4.49	9.56	2.41	2.13	4.89	10.41	2.44	2.16	4.77	10.29	2.37
**13**	1.81	7.85	14.23	1.60	1.72	7.15	12.31	2.31	1.70	7.78	13.2	1.82	1.70	8.03	13.67	1.82
**16**	1.39	1.72	2.39	0.59	1.40	1.74	2.42	1.02	1.39	1.87	2.60	0.80	1.36	1.98	2.69	0.86
**17**	1.41	2.93	4.14	0.58	1.38	2.77	3.81	1.12	1.37	3.02	4.14	0.85	1.33	3.15	4.20	0.85
**19**	1.64	3.91	6.41	1.61	1.61	3.36	5.39	3.81	1.59	3.72	5.91	2.25	1.57	3.69	5.80	2.10

nd: not determined.

**Table 3:** Chromatographic results of the enantioselective separations of selected racemic compounds on photochemically immobilized PMBC-Im CSPs: influence of applied solvent for irradiation. Mobile phase: hexane/2-propanol (9/1, *v*/*v*).

Compound	PMBC-Im G				PMBC-Im F				PMBC-Im C			
	α	k_1_	k_2_	Rs	α	k_1_	k_2_	Rs	α	k_1_	k_2_	Rs
**1**	7.22	1.59	11.50	5.04	7.52	1.40	10.51	6.32	7.05	1.85	13.09	4.53
**2**	1.40	3.63	5.06	1.28	1.34	3.59	4.81	1.51	1.32	4.87	6.41	1.36
**4**	1.00	8.07	8.07	0	1.08	6.93	7.50	0.51	1.05	9.85	10.40	nd
**5**	1.24	8.66	10.80	0.64	1.27	7.40	9.38	0.95	1.23	10.86	13.40	0.73
**7**	1.22	5.23	6.37	0.82	1.17	5.32	6.21	0.76	1.18	7.42	8.76	0.63
**9**	2.21	3.53	7.80	2.76	2.13	3.25	6.93	3.45	2.17	4.80	10.40	3.07
**13**	1.70	6.13	10.50	2.06	1.76	5.62	9.87	2.66	1.72	7.62	13.10	1.96
**16**	1.38	1.36	1.87	0.80	1.34	1.44	1.94	1.04	1.39	1.85	2.56	1.07
**17**	1.36	2.18	2.98	0.86	1.32	2.26	2.98	1.11	1.37	2.91	3.98	0.84
**19**	1.59	2.78	4.43	1.74	1.58	2.62	4.15	2.27	1.61	3.65	5.86	2.44

nd: not determined.

**Table 4:** Chromatographic results of the enantioselective separations of racemates **1**–**19** on photochemically immobilized PMBC-Im E: influence of mobile phase.

Compound	PMBC-S			PMBC-Im E			PMBC-Im E			PMBC-Im E			PMBC-Im E			PMBC-Im E		
	Hexane/2-propanol 9/1			Hexane/2-propanol 9/1			Heptane/Chloroform 90/10			Heptane/Chloroform 75/25			Heptane/Chloroform 67.5/32.5			Heptane/Chloroform 60/40		
	α	k_2_	Rs	α	k_2_	Rs	α	k_2_	Rs	α	k_2_	Rs	α	k_2_	Rs	α	k_2_	Rs
**1**	9.15	16.89	3.71	6.89	13.62	4.37	3.29	3.69	6.36	1.85	0.72	2.56	1.57	0.49	1.56	1.36	0.37	0.80
**2**	1.47	7.82	1.21	1.30	6.30	1.04	1.42	4.07	2.76	1.17	0.66	0.72	1.00	0.40	0.00	1.00	0.30	0.00
**3**	1.37	8.40	0.60	1.30	5.46	2.44	1.31	21.22	3.08	1.23	4.85	2.14	1.21	3.17	1.80	1.19	2.24	1.46
**4**	1.00	14.06	0.00	1.12	10.50	0.86	1.25	30.58	2.00	1.20	5.18	1.82	1.18	3.17	1.54	1.16	2.09	1.16
**5**	1.30	13.01	0.65	1.22	13.82	0.66	1.30	13.89	1.90	1.34	2.16	2.06	1.36	1.41	1.96	1.38	1.01	1.76
**6**	*not soluble*			*not soluble*			1.11	127.82	nd	1.32	13.84	2.60	1.43	6.97	3.16	1.50	4.46	3.30
**7**	1.22	9.35	nd	1.16	8.95	0.54	2.99	49.42	3.70	1.29	20.70	2.62	1.23	11.68	2.18	1.16	7.58	nd
**8**	**1.19**	14.46	nd	1.17	13.00	0.98	1.00	1.00	0.00	1.26	14.72	2.26	1.25	8.05	2.08	1.23	5.27	1.86
**9**	2.53	10.11	2.25	2.16	10.29	2.37	2.11	7.32	5.04	1.57	1.32	2.44	1.38	0.82	1.50	1.27	0.62	0.88
**10**	1.30	8.28	0.50	1.28	8.03	1.68	1.30	10.49	2.16	1.22	1.90	1.52	1.19	1.20	1.10	1.18	0.88	0.74
**11**	*not soluble*			*not soluble*			*not eluted*			3.09	58.45	9.62	2.45	19.90	7.52	2.10	8.77	5.62
**12**	1.00	13.75	0.00	1.00	8.42	0.00	1.18	5.77	nd	1.00	0.82	0.00	1.00	0.49	0.00	1.00	0.36	0.00
**13**	1.81	14.23	1.60	1.70	13.67	1.82	1.89	35.00	4.96	1.68	4.99	4.64	1.60	2.62	3.72	1.48	1.69	2.62
**14**	*not eluted*			*not eluted*			*not eluted*			1.58	59.68	1.74	1.43	22.05	0.92	nd	12.04	nd
**15**	*not eluted*			*not eluted*			*not eluted*			1.23	66.00	2.30	1.24	27.53	2.12	1.25	14.27	nd
**16**	1.39	2.39	0.59	1.36	2.69	0.86	1.98	29.16	5.36	1.65	6.55	4.50	1.54	4.18	3.70	1.43	2.95	2.86
**17**	1.41	4.14	0.58	1.33	4.20	0.85	1.83	52.23	4.92	1.59	10.83	4.62	1.47	6.34	3.70	1.37	4.31	2.78
**18**	**1.13**	5.84	nd	1.09	5.20	nd	1.15	4.91	1.18	1.09	1.04	0.50	1.00	0.67	0.00	1.00	0.50	0.00
**19**	1.64	6.41	1.61	1.57	5.80	2.10	2.02	20.07	7.98	1.76	5.27	5.64	1.65	3.22	4.54	1.55	2.22	3.52

nd: not determined.
